# Strategies to Reduce the Door-to-Device Time in ST-Elevation Myocardial Infarction Patients

**Published:** 2019-01

**Authors:** Mojtaba Salarifar, Javad Askari, Mohammad Saadat, Babak Geraiely, Negar Omid, Hamidreza Poorhosseini, Alireza Amirzadegan, Alimohammad Hajzeinali, Mohammad Alidoosti, Hassan Aghajani, Younes Nozari, Ebrahim Nematipoor

**Affiliations:** *Tehran Heart Center, Tehran University of Medical Sciences, Tehran, Iran. *

**Keywords:** *Myocardial infarction*, *ST elevation myocardial infarction*, *Angioplasty*

## Abstract

**Background:** Performing primary percutaneous coronary intervention (PPCI) in a timely fashion is a crucial part of the management of ST-elevation myocardial infarction (STEMI). We aimed to evaluate the contributing factors to and the etiologies of a prolonged door-to-device (D2D) time.

**Methods:** In 2016, the D2D time was measured in all patients who were treated with PPCI at Tehran Hear Center. The major causes of a prolonged D2D time (>90 min) were determined. The second phase was then started in 2017 by focusing on the determined causes, and direct feedback was given to anyone having contributed to the delayed D2D time. The D2D time was compared between these 2 years.

**Results:** The mean age of the patients was 59.54±11.82 years, and 82.2% of them were men. The median D2D time decreased from 55 minutes (IQR_25-75%_: 40–82) in 2016 to 46 minutes (IQR_25-75%_: 34–70) in 2017 (P<0.001). In the first year, 79.8% of the patients had a D2D time of below 90 minutes; the figure rose to 84.1% of the patients in the second year (P=0.017). The first cause of a prolonged D2D time was missed ST-elevation in the first electrocardiogram by physician or nurse (8.4% of the cases). Along with a declining rate of missed STE to 6.7%, the median D2D time in the missed patients also decreased from 205 minutes to 177 minutes (P=0.011). The rate of ambulance arrival increased from 10.2% to 20.7% of the cases, and the median D2D time also declined from 45 (IQR_25-75%_: 34–55) to 34 (IQR_25-75%_: 25–55) in these patients (P<0.001).

**Conclusion:** Even in the setting of a 24/7 on-site interventionist in the hospital, the dispatch system and prehospital electrocardiograms, along with regular assessment and feedback, may improve the D2D time.

## Introduction

Primary percutaneous coronary intervention (PPCI) is the treatment of choice for ST-elevation myocardial infarction (STEMI). Most developed countries have implemented comprehensive national programs to enhance the coverage of PPCI in a timely fashion.^1^^, ^^2^  There is no better phrase than “time is muscle” to express the importance of time in treating patients with STEMI,^   3 ^ and several studies have found a significant relationship between prolonged ischemic times and poor outcomes.^4^^-^^7^ 

Several metrics such as symptom-to-door (S2D), door-in to door-out, and door-to-device (D2D) times have already been studied to measure the lost time in the chain of events and the management of STEMI. The D2D time, as well as the more recently modified measure, first-medical-contact to device, has been suggested as an indicator of the quality of care in STEMI.^1^ Developed countries have spared no time and effort to lower the D2D time. ^4^^, ^^8^^, ^^9^  The 24/7 program, a national example of such programs, was recently launched in Iran to decrease the delay before PPCI in the treatment of STEMI. However, there are no studies on the efficacy of this program in Iran. 

In the present study, we aimed to investigate the D2D time in patients with STEMI treated in the 24/7 program in Tehran Heart Center (THC). We also evaluated the contributing factors to a prolonged D2D time in this setting.

## Methods

This is a 2-phase study. The first phase was a retrospective cohort on patients with STEMI who underwent PPCI in THC between December 2015 and December 2016 (first year) using the 24/7 registry and the other integrated data banks of THC. In the case of a prolonged D2D time, the patients’ medical records were examined to find the etiologies of the delay. Patients with STEMI who were not candidated for PPCI for any reason, including but not limited to presentation after 48 hours without chest pain or arrhythmia, were excluded. The other exclusion criteria were having prohibitive comorbid conditions, refusing to undergo PPCI, undergoing rescue PCI after thrombolytic administration, and death before PCI. Patients who left the catheterization laboratory (C-Lab) with no intervention (false-positive 24/7 code) were also excluded. The D2D time was not an exclusive criterion per se, and patients with even very long D2D times were included in the analysis. All the patients provided an informed consent. The study was approved by the Ethics Committee of THC. The second phase of the study or the interventional phase was commenced in December 2016. After 1 year of data collection, a preliminary analysis was conducted to identify the major causes of a prolonged D2D time. An integrated program was then started in December 2016 to decrease the D2D time by focusing on the major causes of the delay. A team consisting of the treatment deputy of the hospital, the head of the emergency department (ED), the head nurse of the ED, the head nurse of the C-Lab, and the director of the 24/7 data registry reviewed the medical records of the patients who had prolonged D2D times in monthly meetings to determine the major etiologies of the delay. The team also evaluated a portion of the files of all the STEMI patients to determine whether or not there was compatibility between the information recorded in the 24/7 forms and the information recorded in the system as well as the information in the medical records. A fast-track protocol was developed and communicated to all the staff of the ED and the C-Lab to facilitate the entrance of the patients who were transferred by emergency medical services (EMS) and the dispatch system to the C-Lab without any delay. According to the estimated time of arrival, the C-Lab was reserved for these patients unless a more critical patient required priority. A monthly report was prepared, and corresponding feedback and possible solutions were sent to the individuals who contributed to the prolongation of the D2D time. The D2D time was also evaluated from December 2016 to December 2017 (second year) and was compared with the previous year to assess the success of the integrated program.

**Figure 1:**
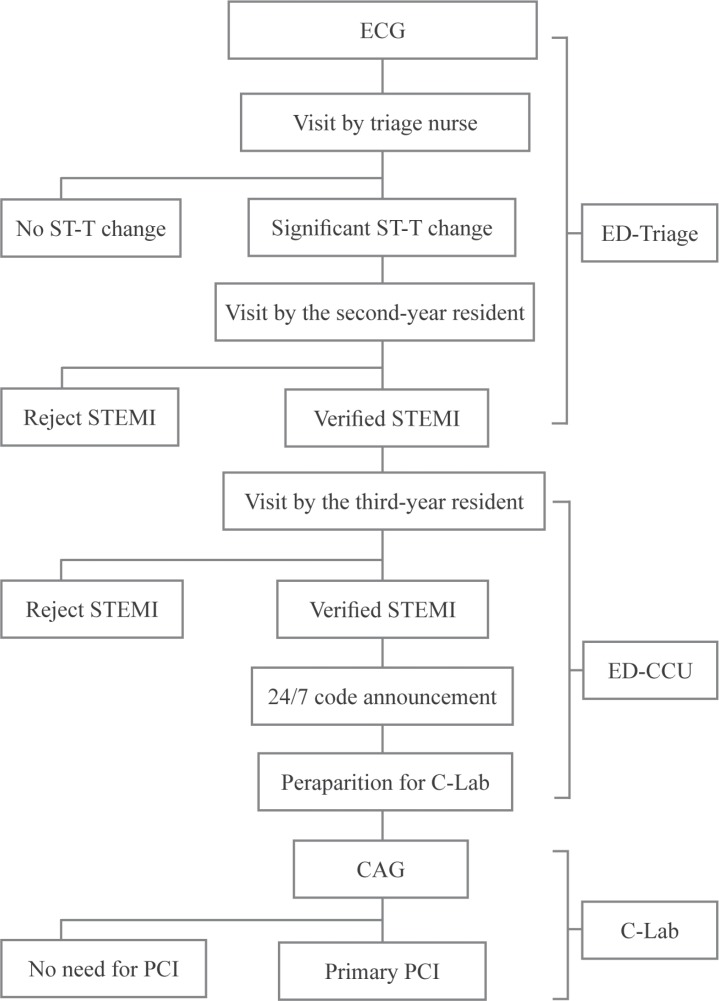
Flowchart of patient admissions to the ED of Tehran Heart Center

ECG, Electrocardiogram; ED, Emergency department; STEMI, ST-elevation myocardial infarction; CCU, Coronary care unit; C-Lab, Catheterization laboratory; CAG, Coronary angiography; PCI, Percutaneous coronary intervention

THC is an academic heart center with more than 400 beds dedicated to both educational and treatment purposes. On average, 80 PPCI procedures are performed in THC per month. Even before the 24/7 national program, PPCI was performed in THC 24 hours a day, 7 days a week (24/7). Figure 1 depicts the process of patient admissions to the ED of THC. Firstly, an electrocardiogram (ECG) is obtained from any patient who comes to the ED complaining of chest pain. This ECG is then evaluated by a trained nurse in the triage room, and if there is any doubt about ST-T changes, it is immediately shown to a second-year cardiology resident always present in the examination room. The patient is visited by the resident straightaway without waiting in the line. If the STE is confirmed by the resident physician, the patient is transferred to the coronary care unit (ED-CCU). Thereafter, the patient is visited by a third-year cardiology resident and if the STEMI is confirmed, the 24/7 code is activated. Next, a telephone call is made to the C-Lab staff and a 24/7 on-site cardiac interventionist to perform PPCI. The patient is dressed in a specially designed lounge suit, and a stat dose of medications, including a loading dose of aspirin, clopidogrel, statins, is administered if required. The patient is subsequently transferred to the C-Lab. A national registration form is fulfilled to record the time of each phase in this process. Patients who are transferred to the hospital by emergency medical services (EMS) have their ECGs obtained at the site, and the activation of the 24/7 code enables them to bypass the early stages of triage and enter the ED-CCU directly.

In the present study, for patients presenting with STEMI, whether they self-transported to the hospital or they were transferred by EMS, the door time was considered the time of arrival at the hospital. For patients who did not initially present with STEMI but developed it during hospitalization, the door time was considered to be the time of STE manifestation. The device time was considered to be the time of guide-wire passage through the lesion. The patients were stratified into 2 groups based on their D2D time: the on-time group, comprising those with a D2D time below 90 minutes ^9^^-^^11^  and the delayed group, consisting of those with a D2D time of equal to or more than 90 minutes. The medical records of the patients who had a D2D time exceeding 90 minutes were thoroughly reviewed by an expert committee to find the major etiologies of the D2D time prolongation. Three working shifts at the ED were defined as 8 AM to 4 PM, 4 PM to 12 AM, and 12 AM to 8 AM. All the recorded variables were measured in compliance with the American College of Cardiology’s key data elements and definitions for measuring the clinical management and outcomes of patients with acute coronary syndromes.^   12 ^

The continuous variables are presented as means±standard deviations (SDs) for those with normal distributions and as medians (IQR_25-75%_) for those without normal distributions. The dichotomous variables are presented as numbers (percentages). Group comparisons for the categorical and continuous variables were performed using the χ^2 ^test, the Mann–Whitney test, or the *t*-test as appropriate. For the categorical comparison, the Fisher exact test was used if at least 20% of the cells had an expected frequency of below 5. The statistical analyses were performed using SPSS, version 21.0 (Armonk, NY: IBM Corp), and P values less than 0.05 were considered statistically significant.

## Results

After consideration of the exclusion criteria, in the first year, 734 patients, including 591 (81.1%) men, underwent PPCI for STEMI in our center. The mean age of the study population was 58.92±11.53 years, ranging from 28 to 94 years. In the next year, 759 patients, including 597 (77.9%) men, were incorporated into the study. The baseline characteristics of these patients are presented in Table 1.

Table 2 shows the characteristics of the STEMI events in terms of the location of Myocardial Infarction (MI), the time of MI, the route of admission, and also the time intervals. The median D2D time and the median pain-to-door time were 55 (IQR_25-75%_: 40–82) minutes and 244 (IQR_25-75%_: 109–565) minutes, respectively. In this year, 586 (79.8%) patients had a D2D time below 90 minutes and 428 (58.3%) had a D2D time below 60 minutes. In the patients who received on-time PPCI (<90 min), the median D2D time was 50 (IQR_25-75%_: 39–62) minutes and this figure was 165 (IQR_25-75%_: 110–225) minutes in the delayed group. Cardiovascular risk factors, age or marital status or education level, the location of MI, and the time and route of arrival were not significantly different between the 2 groups. 

**Figure 2:**
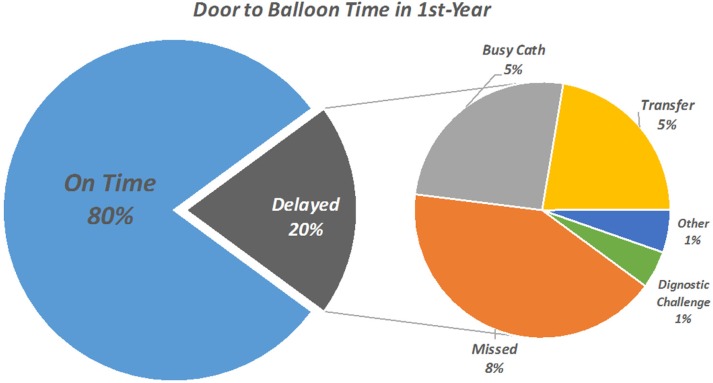
Door-to-balloon time in the first year

Among the patients with a D2D time of more than 90 minutes, undiagnosed STE in the first ECG by physician or nurse (either due to a subtle STE or the presence of bundle branch block or paced rhythms) caused a delay in 62 (41.8%) patients. Logistic constraints, including C-Lab occupancy (25.7%) and deficiencies in the transportation of the patient from the ED to the C-Lab (22.3%), were 2 other main causes of a prolonged D2D time (Figure 2). 

The median D2D time was 165 (IQR_25-75%_: 110–225) minutes in the delayed group. However, in the patients who were delayed due to undiagnosed STE, the median D2D time was 205 (IQR_25-75%_: 146–249) minutes. This figure was 128 (IQR_25-75%_: 102–206) and 134 (IQR_25-75%_: 107–193) minutes, correspondingly, in the patients who experienced delay due to a busy C-Lab and transportation problems (Table 3). For each patient, the total minutes exceeding 90 minutes before the conduct of PCI were calculated. From a total of 13 559 minutes, medical errors were responsible for the loss of 7838 (57.7%) minutes, the occupancy of the C-Lab accounted for the loss of 2615 (19.4%) minutes, and transportation issues were culpable for the loss of 2104 (15.5%) minutes (Table 3). 

**Figure 3:**
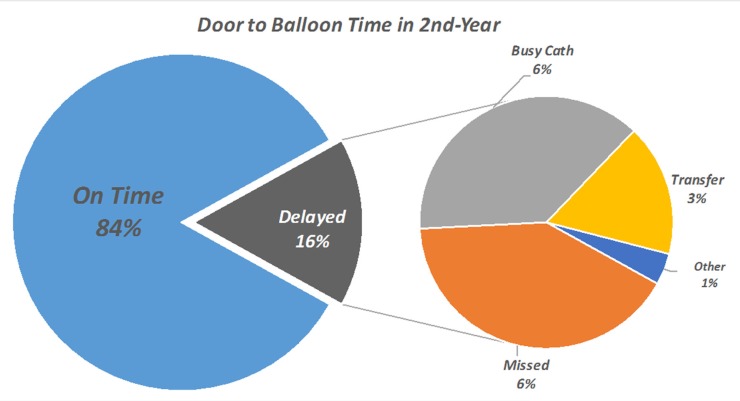
Door-to-balloon time in the second year.

**Figure 4:**
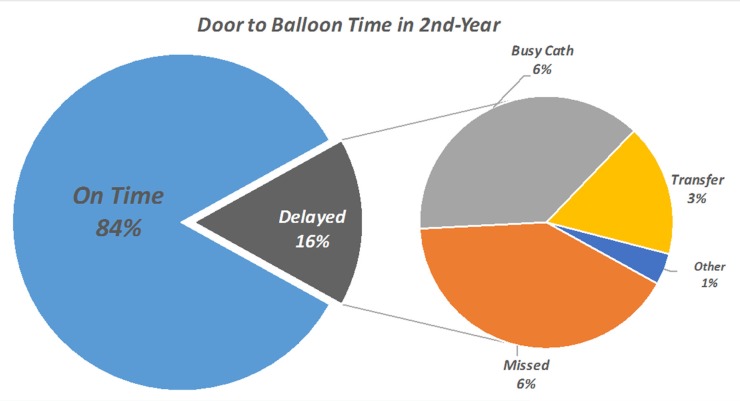
Median of the door-to-device time in the first and second years

In the next year, the median D2D time decreased from 55 (40–82) minutes to 46 (IQR_25-75%_: 34–70) minutes (P<0.001), which showed a significant reduction in the D2D time in the second year. The percentage of the patients with a D2D time below 90 minutes and below 60 minutes also rose to 84.1% and 69.7%, respectively (P=0.017 and P=0.007). In the second year, missed STE remained the major cause of a prolonged D2D time (Figure 3), but its frequency dropped from 8.44% to 6.66% (P=0.184). Along with the reduction in the rate of missed STE, the median D2D time in the missed patients also decreased from 205 (IQR_25-75%_: 146–249) minutes to 177 (IQR_25-75%_: 120–232) minutes (P=0.011) (Figure 4). These 2 changes together caused a reduction in the total minutes lost due to missed STE from 57.7% to 49.6%, which showed a 24% reduction (Table 3). The configuration of the arrival route was also changed in the second year (Table 4) insofar as the number of the patients transferred to the hospital by EMS (either direct or indirect) rose from 10.2% to 20.7%. As is evident, the median D2D time also significantly declined from 45 (IQR_25-75%_: 34–55) and 50 (IQR_25-75%_: 41–60) minutes to 34 (IQR_25-75%_: 25–55) and 33 (IQR_25-75%_: 25–50) minutes in these patients, resulting in a decreased overall D2D time in the second year.

C-Lab, Catheterization laboratory; STE, ST elevation

C-Lab, Catheterization laboratory; STE, ST elevation

C-Lab, Catheterization laboratory; STE, ST elevation

**Table 1:** Comparison of the baseline characteristics of the patients between the first and second years (N=1493)*

	**First Year**	**Second Year**	**P**
	**(N=734)**	**(N=759)**	
**Age (y)**	58.92±11.53	60.06±12.04	0.208
**Male **	595 (81.1)	633 (83.4)	0.250
**ST-Elevation Leads**			
** Precordial**	324 (46.2)	355 (46.3)	0.943
** Lateral**	103 (14.7)	112 (14.6)	0.978
** Inferior**	311 (44.3)	334 (43.6)	0.788
** Posterior**	76 (10.8)	79 (10.3)	0.749
**Right side**	68 (9.7)	91 (11.9)	0.177
** BBB-MI**	5 (0.7)	3 (0.4)	0.495
**Culprit Vessel**			
** Left main**	3 (0.4)	4 (0.5)	1.000
** LAD**	324 (46.2)	358 (46.7)	0.823
** LCX**	73 (10.4)	86 (11.2)	0.620
** RCA**	205 (29.2)	227 (29.6)	0.856
** SVG**	8 (1.1)	11 (1.4)	0.616
** Diagonal**	23 (3.3)	34 (4.4)	0.250
** OM**	33 (4.7)	52 (6.8)	0.087
** Ramus**	2 (0.3)	8 (1.0)	0.112
** PDA**	16 (2.3)	18 (2.3)	0.928
** PLB**	19 (2.7)	15 (2.0)	0.341
**Season **			
** Spring**	125 (17.3)	185 (25.6)	0.203
** Summer**	199 (27.5)	156 (21.5)	0.871
** Fall**	293 (40.5)	219 (30.2)	0.265
** Winter**	107 (14.8)	164 (22.7)	0.681
**Route of Arrival**			
** Ambulance-PPCH**	35 (5.6)	65 (10.7)	<0.001
** Ambulance-NPPCH**	29 (4.6)	61 (10.0)	<0.001
** Self-transported**	550 (88.0)	469 (76.9)	<0.001
** Admitted **	11 (1.8)	15 (2.5)	0.570
**Work Shifts of ED**			
** One**	322 (43.9)	308 (40.6)	0.211
** Two**	227 (30.9)	251 (33.0)	0.324
** Three**	185 (25.2)	200 (26.4)	0.547

BBB-MI, Bundle branch block myocardial infarction; LAD, Left anterior descending artery; LCX, Left circumflex artery; RCA, Right coronary artery; SVG, Saphenous vein graft, OM, Obtuse marginal; PDA, Posterior descending artery; PLB, Posterior left ventricular branch; PPCH, Primary percutaneous coronary intervention capable hospital; NPPCI, Non–primary percutaneous coronary intervention capable hospital; ED, Emergency department; Work shifts of ED, Work shift of the emergency department as Shift 1: 8 Am to 4 PM, Shift 2: 4 PM to 12 AM, and Shift 3: 12 AM to 8 AM

* Data are presented as n (%) or mean±SD.

**Table 2:** Index-event characteristics in the study population in the first year (N=734)*

	**On-time**	**Delayed**	**P** **	**Median (IQR **_25-75%_ **) of ** **D2D time**	**P** ***
	**586 (79.8)**	**148 (20.1)**			
**Pain-to-door (min)**	230 (100-538)	322 (124-615)	<0.001		
**Door-to-device (min)**	50 (39-62)	165 (110-225)	<0.001		
**ST-Elevation Leads** *					0.041
** Precordial**	268 (82.7)	56 (17.3)	0.118	55 (40-75)	
** Lateral**	88 (85.5)	15 (14.5)	0.153	54 (40-75)	
** Inferior**	246 (79.0)	65 (21.0)	0.507	53 (40-85)	
** Posterior**	64 (84.2)	12 (15.8)	0.352	55 (43-80)	
** Right side**	58 (85.3)	10 (14.7)	0.271	50 (39-64)	
** BBB-MI**	2 (40.0)	3 (60.0)	0.027	135 (48-216)	
**Culprit Vessel**					0.463
** Left main**	2 (66.7)	1 (33.3)	0.481	56 (-)	
** LAD**	267 (82.4)	57 (17.6)	0.177	55 (40-76)	
** LCX**	63 (86.3)	10 (13.7)	0.169	56 (40-76)	
** RCA**	161 (78.5)	44 (21.5)	0.483	55 (40-85)	
** SVG**	7 (87.5)	1 (12.5)	0.614	47 (37-59)	
** Diagonal**	19 (82.6)	4 (17.34)	0.815	59 (40-85)	
** OM**	26 (78.8)	7 (21.2)	0.961	62 (44-87)	
** Ramus**	0	2 (100)	0.038	179 (-)	
** PDA**	10 (62.5)	6 (37.5)	0.078	75 (42-118)	
** PLB**	13 (68.4)	6 (31.6)	0.193	68 (44-126)	
**Season **					0.115
** Spring**	105 (84.0)	20 (16.0)	0.204	53 (40-70)	
** Summer**	158 (79.4)	41 (20.6)	0.872	55 (41-87)	
** Fall**	228 (77.8)	65 (22.2)	0.265	56 (40-81)	
** Winter**	87 (81.3)	20 (18.7)	0.681	55 (44-82)	
**Route of Arrival**					0.016
** Ambulance-PPCH**	31 (81.9)	4 (10.9)	0.138	45 (34-55)	
** Ambulance-NPPCH**	27 (93.1)	2 (6.9)	0.084	50 (41-60)	
** Self-transported**	437 (79.5)	113 (20.5)	0.357	56 (42-85)	
** Admitted patient**	7 (63.6)	4 (36.4)	0.158	75 (34-160)	
**Work Shifts of ED**					0.221
** One**	267 (82.9)	55 (17.1)	0.063	55 (40-75)	
** Two**	174 (76.7)	53 (23.3)	0.153	57 (42-86)	
** Three**	145 (78.4)	40 (21.6)	0.571	55 (40-83)	

BBB- MI, Bundle branch block myocardial infarction; LAD, Left anterior descending artery; LCX, Left circumflex artery; RCA, Right coronary artery; SVG, Saphenous vein graft, OM, Obtuse marginal; PDA, Posterior descending artery; PLB, Posterior left ventricular branch; PPCH, Primary percutaneous coronary intervention capable hospital; NPPCI, Non–primary percutaneous coronary intervention capable hospital; ED, Emergency Department; Work shifts of ED, Work shift of the emergency department as Shift 1: 8 Am to 4 PM, Shift 2: 4 PM to 12 AM, and Shift 3: 12 AM to 8 AM

* Data are presented as n (%) or median (IQR _**25-75%**_).

** P value for the comparison of the percentage of each contributing factor between the 2 groups (on time and delayed)

*** P value for the comparison of the median D2D time between the subgroups

**Table 3:** Etiologies of delay in the patients with a D2D time of more than 90 minutes in the first and second years

	First Year (N=148)			Second Year (N=122)			P*	P**
	No. of Delayed Patients (%)	Median (IQR _25-75%_ of D2D time	Lost Minutes (% of total)	No. of Delayed Patients (%)	Median (IQR _25-75%_) of D2D time	Lost Minutes (% of total)		
Undiagnosed STE	62 (41.8)	205 (146-249)	7838 (57.7)	51 (41.7)	177 (120-232)	5181 (49.6)	0.184	0.011
Busy catheterization laboratory	38 (25.7)	128 (102-206)	2615 (19.4)	47 (38.5)	130 (101-177)	3452 (33.0)	0.423	0.942
Transportation deficiency	33 (22.3)	134 (107-193)	2104 (15.5)	21 (17.2)	130 (104-174)	1629 (15.5)	0.076	0.826
Diagnostic challenge	4 (2.7)	140 (102-183)	207 (1.5)	-	-	-	0.068	-
Arrest and CPR in ED	4 (2.7)	189 (153-276)	465 (3.4)	-	-	-	0.068	-
Senility and severe comorbidities	3 (2.0)	165 (-)	188 (1.4)	1 (0.9)	284 (-)	194 (1.8)	0.364	0.512
Obtaining informed consent	2 (1.4)	146 (-)	112 (0.8)	-	-	-	0.124	-
Difficult angioplasty	2 (1.4)	105 (-)	30 (0.3)	2 (1.7)	95 (-)	10 (0.1)	0.498	0.746

D2D, Door-to-device; STE, ST elevation; CPR, Cardiopulmonary resuscitation; ED, Emergency department

* P value for the comparison of the percentage of each delay etiology between the 2 years

** P value for the comparison of the median D2D time between the subgroups in the 2 years

**Table 4:** Evaluation of the potential role of the route of arrival in reducing the D2D time in the first and second years

	**First Year (N=734)**		**Second Year (N=759)**		**P** *
	**Number (%)**	**Median D2D Time** **(IQR **_25-75%_ **)**	**Number (%)**	**Median D2D Time ** **(IQR **_25-75%_ **)**	
**Ambulance-PPCH**	35 (5.6)	45 (34-55)	65 (10.7)	34 (25-55)	<0.001
**Ambulance-NPPCH**	29 (4.6)	50 (41-60)	61 (10.0)	33 (25-50)	<0.001
**Self-transported**	550 (88.0)	56 (42-85)	469 (76.9)	50 (36-75)	0.026
**Admitted patient**	11 (1.8)	75 (34-160)	15 (2.5)	50 (38-85)	<0.001

D2D, Door to device; PPCH, Primary percutaneous coronary intervention-capable hospital; NPPCI, Non–primary percutaneous coronary intervention-capable hospital

* P value for the comparison of the median D2D time between the subgroups in the 2 years

## Discussion

The present study is the first brief report on the D2D time status in one of the largest heart centers in Iran. We found that the median D2D time and the percentage of the patients with a D2D time below 90 minutes were 55 minutes and 79.8% in 2016 and 46 minutes and 84.1% in 2017, respectively. The major causes of a prolonged D2D time were undiagnosed STE by physician or nurse, unavailable C-Lab, and transportation. Our study showed that these delays could be reduced by implementing integrated evaluations and feedback programs and the enhancement of the EMS-based transportation system. Shortening the D2D time is an important intervention. Indeed, several studies have demonstrated that the prognosis of patients with STEMI may be largely affected by the D2D time ^3^^-^^6^  and this parameter is generally regarded as a measure of quality control in hospitals.^   1 ^

There are several national and local studies on the D2D time or its predecessor, the door-to-balloon time, in developed and developing countries. The D2D time in the United States was reported to be 111 minutes in 1994, but it continuously declined to 79 minutes in 2006^   13 ^ and 64 minutes in 2010.^   14 ^ The D2D time was reported to be 64 minutes in the Netherlands in 2012,^   15 ^ 92 minutes in Japan in 2013,^   16 ^ and 65 minutes in Australia in 2014.^   17 ^ In developing countries, the D2D time was reported to be 75 minutes in a single center in India.^   18 ^ The D2D time decreased from 155 minutes to 73 minutes in Kazakhstan over a time period between 2012 and 2015.^   19 ^ The D2D time in our center appears to be comparable with the best results reported from developed countries and far shorter than the time in other developing countries. It is worthy of note that most studies have excluded some patients and, as such, have underestimated their reported time. ^20^^-^^22^  For example, a large number of studies have excluded patients with very long D2D times (eg, >3 h) ^5^^, ^^8^^, ^^14^^-^^16^  and some studies have excluded high-risk patients such as those with cardiogenic shock, cardiac arrest, and diagnostic challenges. ^14^^, ^^18^  Khot et al.^21^ suggested that all patients undergoing PPCI be included for a real estimation of the D2D time and no exclusion be applied based on the length of delay and the medical condition of the patients, which is exactly what we did in the current study.

Limited data are available about the etiology of delay in PPCI. For all the studies that have evaluated the contributing factors to the D2D time prolongation, few studies have directly addressed the causes of delay, especially system-related causes in a hospital setting. Several differences in the findings between our study and other studies merit consideration. In a study conducted by Cotoni et al.,^   22 ^ difficulties in obtaining a vascular access, cardiac arrest and resuscitation, and difficult PCI were reported as the major causes of the delay among non-system related etiologies. However, the actual proportions of the system-related and non-system related delays were not reported. In our case, these causes were able to explain the delay only in 6 patients, accounting for 4.1% of the total figure. In a study by Victor et al.,^   18 ^ financial issues and informed consent were reported as 2 major causes of delay. Nevertheless, these 2 parameters were not even considered in our study. According to the national insurance system in our country, no payment is required before the procedure, so financial issues are not discussed with patient or family before PPCI. In contrast to our study, Victor and colleagues found a hospital-related delay in 20% of their cases, while the majority of the delayed cases were patient-related. There are some reports suggesting that aggressive attempts to shorten the D2D time not only have some adverse consequences, including false C-Lab activation and increased cost of care, but also do not necessarily improve the outcomes.^8^^, ^^23^^, ^^24^  For this reason, 60 minutes was suggested as an optimal median D2D time. 

There are several suggested strategies to reduce the D2D time ^4^^, ^^11^^, ^^25^^-^^28^  and centers should choose one or some of them based on local considerations. Some of these strategies require investment in infrastructure such as providing prehospital ECGs^25^^, ^^24^ and on-site cardiac interventionists, ^10^^, ^^11^  while some other could be applied in most hospitals with limited budgets by modifying the internal protocols such as the activation of the STEMI code by emergency physicians^   26 ^ and the direct transfer of patients to the C-Lab and bypassing the ED.^   11 ^ Finally, there are options like continuous monitoring and feedback programs,^   28 ^ which do not even require time and effort to draw up protocols and only require administrative approval.^   27 ^

As was mentioned above, in our study, the first cause of delay was medical error (misinterpretation of the first ECG), followed by structural insufficiencies. In our center, if STE is missed in the first ECG, the patient is kept under observation and most of these missed cases are later discovered after troponin tests return positive. This causes delay in this group as it generally takes troponin results at least 2 hours to come in. Alongside improving structural facilities, an effective intervention requires educational programs for both residents and nurses. Although we believe that patient visit by a resident of cardiology is preferable to that by an emergency physician, it could never be comparable to that by an experienced staff cardiologist. Our study showed that even in a high-volume center such as THC, continuous monitoring of the D2D time and direct feedback to persons after errors could significantly reduce the D2D time. Accelerating the laboratory measurement of troponin might be considered another intervention to further decrease the D2D time. Although it may not reduce the proportion of patients with a D2D time beyond 90 minutes, it could probably decrease the lost time in undiagnosed patients. 

It does not necessarily mean that the difference between the 2 stages of our study is totally attributable to our intervention. The physician residents at the ED were rotated in these 2 years and the house staff grew more familiar with the 24/7 program over time. The first year of our study was conducted at a time when prehospital ECGs and the dispatch center had not been widely implemented in Tehran. Only recently have prehospital ECGs been sent to the hospital and the C-Lab team has been activated before patient arrival. The significant reduction in the D2D time of these patients showed that the dispatch system and prehospital ECGs could be beneficial, even in the setting of a permanent resident interventionist. Despite all the efforts that have been made to reduce the D2D time, it should be admitted that the ED-based system has its own limitations, which preclude a significant reduction in the D2D time. It appears that the most important way to significantly lower the D2D time in all hospitals is to expand the coverage of the dispatch system and the obtaining and transferring of an ECG to the hospital to activate the C-Lab unit.

Our study is the first large-scale report on the D2D time status in Iran in the post-24/7 program era. The major limitation of this study is that our results cannot be extended to many other centers and hospitals. Accordingly, our findings should not be interpreted as a national or large-scale report. THC and many other specialized single-disciplinary hospitals that manage patients just in a single field of medicine have a specific type of infrastructure that could not be applied to many other general hospitals tasked with the management of patients with all manner of complaints. Our center enjoys several features that help shorten the D2D time: not only is it a high turn-over specialized center but also it boasts on-site resident interventionists and direct patient transfer to the C-Lab. ^10^^, ^^11^^, ^^26^^-^^29^  These characteristics could not be easily replicated in other general hospitals. Nevertheless, we believe that this report could serve as a reference for other centers.

## Conclusion

Our study showed that the D2D time was acceptable in THC in 2016 and medical errors were the most prevalent cause of the D2D time prolongation in that they accounted for more than half of the total minutes lost. Despite improvements in the D2D time in all the categories of patients, it should be noted that the ED-based system has limitations and even in the setting of the permanent residency of interventionists in the hospital, the dispatch system and prehospital ECGs may significantly improve the D2D time.

## Notes:

***This paper should be cited as: ***Salarifar M, Askari J, Saadat M, Geraiely B, Omid N, Poorhosseini H, Amirzadegan A, Hajzeinali A, Alidoosti M, Aghajani H, Nozari Y, Nematipoor E. Strategies to Reduce the Door-to-Device Time in ST-Elevation Myocardial Infarction Patients. J Teh Univ Heart Ctr 2019;14(1):18-27.
